# Supplementary material for: Mild antecedent COVID-19 associated with symptom-specific post-acute sequelae
Source: PLoS One. 2023 Jul 10;18(7):e0288391. doi: 10.1371/journal.pone.0288391 (PMC10332615; doi:10.1371/journal.pone.0288391)
Supplement: S1 Table — (DOCX) [file pone.0288391.s001.docx]

**Supplementary Table 1. Post-acute Sequelae of SARS-CoV-2 Review of Symptoms**

|  | |
| --- | --- |
| General  Fatigue or low energy  Weight loss  Fevers or chills  Flushing or the sudden sensation of feeling hot  Insomnia or difficulty getting to sleep  Excessive sleeping | Gastroenterology  Heartburn or acid reflux  Nausea and/or vomiting  Diarrhea |
| Neurocognitive  Headaches  Difficulty remembering things  Forgetting words  Feeling like your thinking is slow  Difficulty concentrating  Hearing loss  Loss of smell  Loss of taste  Ringing in your ear  Dizziness | Musculoskeletal  Muscle pain  Joint pains  Weakness in your arms or legs |
| Cardiovascular  Fast or pounding heart beats, palpitations  Chest pain | Dermatology  Rash  Hair loss |
| Pulmonology  Shortness of breath  Cough  Wheezing | Psychiatry  Depression  Anxiety |
