# Supplementary material for: Mild antecedent COVID-19 associated with symptom-specific post-acute sequelae
Source: PLoS One. 2023 Jul 10;18(7):e0288391. doi: 10.1371/journal.pone.0288391 (PMC10332615; doi:10.1371/journal.pone.0288391)
Supplement: S2 Table — (DOCX) [file pone.0288391.s002.docx]

**Supplementary Table 2. Multivariable Logistic Regression Analysis of PASC Symptoms by COVID-19 Variant – Alpha vs Delta Variant**

|  |  | **Acute COVID-19 Severity** | |  |  |
| --- | --- | --- | --- | --- | --- |
| **Symptoms and Outcomes** | **All participants** | **Alpha** | **Delta** | **OR (95% CI)** | **p-value** |
|  | **N= 332** | **N=304**  **Col (%)** | **N=28**  **Col (%)** |  |  |
| Dyspnea | 236 (71) | 153 (71) | 20 (71) | 0.64 (0.25-1.64) | 0.35 |
| Fatigue | 199 (60) | 184 (61) | 14 (50) | 0.79 (0.34-1.86) | 0.59 |
| Cognitive Impairment | 156 (47) | 144 480) | 12 (43) | 0.58 (0.25-1.35) | 0.21 |
| Dizziness | 73 (22) | 66 (22) | 7 (25) | 1.16 (0.45-3.03) | 0.76 |
| Headache | 69 (21) | 64 (21) | 5 (18) | 1.09 (0.36-3.30) | 0.88 |
| Cough | 98 (30) | 93 (31) | 4 (14) | 2.57 (0.82-8.10) | 0.11 |
| Muscle pain | 61 (18) | 52 (17) | 7 (25) | 0.97 (0.36-2.61) | 0.95 |
| Anxiety | 60 (18) | 52 (17) | 7 (25) | 0.36 (0.13-0.98)* | 0.047* |
| Depression | 52 (16) | 48 (16) | 4 (14) | 0.89 (0.32-2.48) | 0.82 |
| Joint pain | 53 (16) | 50 (17) | 3 (11) | 0.62 (0.20-1.96) | 0.42 |
| Palpitations | 53 (16) | 51 (17) | 2 (7) | 2.45 (0.52-11.5) | 0.25 |
| Weakness | 43 (13) | 38 (13) | 5 (18) | 0.95 (0.31-2.92) | 0.92 |
| Sleep disturbances | 40 (12) | 34 (11) | 6 (21) | 0.39 (0.14-1.09) | 0.07 |
| Anosmia | 38 (11) | 35 (12) | 3 (11) | 1.76 (0.43-7.10)* | 0.43 |
| Dysgeusia | 50 (15) | 47 (16) | 3 (11) | 1.64 (0.43-6.34) | 0.47 |
| PROMIS Dyspnea >1.5 SD | 9/253 (17) | 8 (3) | 1 (5) | 0.26 (0.04-1.70) | 0.16 |
| PROMIS Fatigue >1.5 SD | 75/263 (29) | 69 (29) | 6 (27) | 0.57 (0.21-1.58) | 0.28 |
| PROMIS Cognitive >1.5 SD | 59/259 (23) | 53 (22) | 6 (27) | 0.37 (0.13-1.07) | 0.07 |
| PHQ-9 ≥10 | 84/263 (32) | 82 (28) | 2 (5) | 0.58 (0.27-1.24)* | 0.16 |
| GAD-7 ≥10 | 53/127 (42) | 46 (15) | 7 (25) | 1.88 (0.77-4.63) | 0.17 |

*P<0.05 statistically significant
