# Supplementary material for: Mild antecedent COVID-19 associated with symptom-specific post-acute sequelae
Source: PLoS One. 2023 Jul 10;18(7):e0288391. doi: 10.1371/journal.pone.0288391 (PMC10332615; doi:10.1371/journal.pone.0288391)
Supplement: S3 Table — (DOCX) [file pone.0288391.s003.docx]

**Supplementary Table 3. Multivariable Logistic Regression Analysis of PASC Symptoms by Acute COVID-19 Severity – Critical vs Non-Critical**

|  |  | **Acute COVID-19 Severity** | |  |  |
| --- | --- | --- | --- | --- | --- |
| **Symptoms and Outcomes** | **All participants** | **Non-Critical** | **Critical** | **OR (95% CI)** | **p-value** |
|  | **N= 332** | **N=292**  **Col (%)** | **N=40**  **Col (%)** |  |  |
| Dyspnea | 236 (71) | 203 (70) | 33 (83) | 2.71 (1.07-6.88)* | 0.04* |
| Fatigue | 199 (60) | 180 (62) | 19 (48) | 0.61 (0.29-1.27) | 0.19 |
| Cognitive Impairment | 156 (47) | 145 (50) | 11 (28) | 0.35 (0.16-0.80)* | 0.01* |
| Dizziness | 73 (22) | 69 (24) | 4 (10) | 0.46 (0.15-1.39) | 0.62 |
| Headache | 69 (21) | 64 (22) | 5 (13) | 0.64 (0.22-1.85) | 0.41 |
| Cough | 98 (30) | 80 (27) | 18 (45) | 1.99 (0.95-4.17) | 0.07 |
| Muscle pain | 61 (18) | 55 (19) | 6 (15) | 1.09 (0.41-2.92) | 0.86 |
| Anxiety | 60 (18) | 56 (19) | 4 (10) | 0.63 (0.21-1.95) | 0.42 |
| Depression | 52 (16) | 46 (16) | 6 (15) | 0.91 (0.32-2.62) | 0.86 |
| Joint pain | 53 (16) | 52 (18) | 1 (3) | 0.12 (0.02-0.96)* | 0.046* |
| Palpitations | 53 (16) | 49 (17) | 4 (10) | 0.69 (0.20-2.35) | 0.55 |
| Weakness | 43 (13) | 37 (13) | 6 (15) | 1.17 (0.42-3.25) | 0.77 |
| Sleep disturbances | 40 (12) | 36 (12) | 4 (10) | 0.87 (0.27-2.74) | 0.81 |
| Anosmia | 38 (11) | 31 (11) | 7 (18) | 3.08 (1.10-8.67)* | 0.03* |
| Dysgeusia | 50 (15) | 43 (15) | 7 (18) | 1.62 (0.61-4.28) | 0.33 |
| PROMIS Dyspnea >1.5 SD | 9/253 (4) | 7 (3) | 2 (6) | 2.39 (0.36-15.9) | 0.37 |
| PROMIS Fatigue >1.5 SD | 75/263 (29) | 73 (32) | 2 (6) | 0.16 (0.03-0.74)* | 0.02* |
| PROMIS Cognitive >1.5 SD | 59/259 (23) | 57 (25) | 2 (6) | 0.13 (0.02-1.01) | 0.05 |
| PHQ-9 ≥10 | 84/263 (34) | 82 (28) | 2 (6) | 0.44 (0.21-0.93)* | 0.03* |
| GAD-7 ≥10 | 53/127 (42) | 50 (17) | 3 (8) | 1.49 (0.63-3.52) | 0.36 |

*P<0.05 statistically significant
