# Supplementary material for: Mild antecedent COVID-19 associated with symptom-specific post-acute sequelae
Source: PLoS One. 2023 Jul 10;18(7):e0288391. doi: 10.1371/journal.pone.0288391 (PMC10332615; doi:10.1371/journal.pone.0288391)
Supplement: Attachment — Submitted filename: Mild_COVID-19_associated_with_PASC_reviewcomment.docx [file pone.0288391.s004.docx]

Mild antecedent COVID-19 associated with symptom-specific post-acute sequelae

Tiffany A. Walker, MD^1,2^, Alex D. Truong, MD, MPH^3^, Aerica Summers, MD, MPH^2^, Adviteeya N. Dixit, MD^3^, Felicia C. Goldstein, PhD^4^, Ihab Hajjar, MD^1,4^, Melvin R. Echols, MD^5^, Matthew C. Woodruff, MD^6^, Erica D. Lee, PhD^7^, Seema Tekwani, MD^3^, Kelley Carroll, MD^2^, Ignacio Sanz, MD^6^, F. Eun-Hyung Lee, MD^3^, Jenny E. Han, MD, MSc ^2,3^

^1^Department of Medicine, Division of General Internal Medicine, Emory University, Atlanta, GA, 30322, USA

^2^Grady Post-COVID Clinic, Grady Memorial Hospital, Atlanta, GA, 30303, USA

^3^Department of Medicine, Division of Pulmonary, Allergy, Critical Care and Sleep Medicine, Emory University, Atlanta, GA, 30322, USA

^4^Department of Neurology, Emory University School of Medicine, Atlanta, Georgia, 30322, USA

^5^Department of Cardiology, Morehouse School of Medicine, Atlanta, Georgia, 30303, USA

^6^Department of Medicine, Division of Rheumatology, Lowance Center for Human Immunology, Emory University, Atlanta, GA, 30322, USA

^7^Department of Psychiatry and Behavioral Sciences, Emory University School of Medicine, Atlanta, Georgia, 30322, USA

Short title: Mild COVID-19 and post-acute sequelae

Key words: COVID-19, SARS-CoV-2, Post-acute sequelae of SARS-CoV-2 (PASC), antiviral, remdesivir

Abstract word count: 242

Manuscript text word count: 2894

Corresponding author:

Tiffany A. Walker, 49 Jesse Hill Jr Dr. SE Atlanta, GA 30303, Emory University School of Medicine

Fax number: 404-778-1601, Contact number: 678-431-8075, email: [Twalk25@emory.edu](mailto:Twalk25@emory.edu)

**Abstract**

Background

The impact of COVID-19 severity on development of long-term sequelae remains unclear, and symptom courses are not well defined.

Methods

This ambidirectional cohort study recruited adults with new or worsening symptoms lasting ≥3 weeks from confirmed SARS-CoV-2 infection between August 2020–December 2021. COVID-19 severity was defined as severe for those requiring hospitalization and mild for those not. Symptoms were collected using standardized questionnaires. Multivariable logistical regression estimated odds ratios (OR) and 95% confidence intervals (CI) for associations between clinical variables and symptoms.

Results

Of 332 participants enrolled, median age was 52 years (IQR 42-62), 233 (70%) were female, and 172 (52%) were African American. Antecedent COVID-19 was mild in 171 (52%) and severe in 161 (48%). In adjusted models relative to severe cases, mild COVID-19 was associated with greater odds of fatigue (OR:1.83, CI:1.01-3.31), subjective cognitive impairment (OR:2.76, CI:1.53-5.00), headaches (OR:2.15, CI:1.05-4.44), and dizziness (OR:2.41, CI:1.18-4.92). Remdesivir treatment was associated with less fatigue (OR:0.47, CI:0.26-0.86) and fewer participants scoring >1.5 SD on PROMIS Cognitive scales (OR:0.43, CI:0.20-0.92). Fatigue and subjective cognitive impairment prevalence was higher 3-6 months after COVID-19 and persisted (fatigue OR:3.29, CI:2.08-5.20; cognitive OR:2.62, CI:1.67-4.11). Headache was highest at 9-12 months (OR:5.80, CI:1.94-17.3).

Conclusions

Mild antecedent COVID-19 was associated with highly prevalent symptoms, and those treated with remdesivir developed less fatigue and cognitive impairment. Sequelae had a delayed peak, ranging 3-12 months post infection, and many did not improve over time, underscoring the importance of targeted preventative measures.

**Background**

The coronavirus disease 2019 (COVID-19) has resulted in over 600 million cases globally and resulted in greater than 6 million deaths (1). Although there are numerous publications describing the wide range of acute phase manifestations (2), there are more limited data on post-acute sequelae of SARS-CoV-2 (PASC). Common symptoms include fatigue, dyspnea, cognitive impairment, and pain syndromes, but can span multiple organ systems (3-6). While the incidence of PASC is unknown, symptoms lasting ≥ 3 months have been reported in 49-73% of COVID-19 survivors in prospective cohort studies (3, 5, 7-9). PASC can significantly impair quality of life (3, 6, 7, 9), and in many, debilitating symptoms can persist for over a year with an unclear timeline for symptom resolution (3, 7, 10).

Identification of PASC risk factors and clinical course is essential to guiding public health education and COVID-19 mitigation strategies. Although early studies showed higher rates of persistent symptoms in patients who experienced severe SARS-CoV-2 infections, the impact of antecedent COVID-19 severity on PASC development remains unclear as further studies have shown conflicting findings (3, 7, 11-14). Delineation of PASC symptom trajectories may provide insight into potential pathophysiological processes. Given the heterogeneity of PASC symptoms and presentations, there are data to support distinct phenotypes that may result from different underlying mechanisms (6).

We identified risk factors associated with persistent symptoms in adults presenting to our PASC clinics, including association with acute COVID-19 severity and treatment. Additionally, although PASC has been defined by the Delphi consensus as symptoms usually lasting 3 months from COVID-19 onset (15), little is known about underlying mechanisms driving PASC phenotypes and the timeline in which these processes take course. Therefore, we characterized prevalent symptom profiles across 3-month intervals beginning 3 weeks from acute COVID-19 onset to inform symptom courses in our population.

**Methods**

*Study Population and Design*

This ambidirectional cohort study consecutively recruited adults aged ≥18 years with new or worsening symptoms lasting ≥3 weeks from confirmed SARS-CoV-2 test from two academic PASC clinics at Emory University and Grady Memorial Hospital in Atlanta, GA between August 2020–December 2021. We excluded participants who had 1) no persistent symptoms ≥3 weeks from disease onset, 2) no documentation of positive SARS-CoV-2 RNA or antigen from nasopharyngeal swab. Due to the high volume of patients referred to our PASC clinics during the study period, standardized follow-up could not be consistently accommodated. For the purposes of this study, participants are only represented at one time point. Ethics approval was obtained from Emory University IRB prior to study initiation, and written consent was obtained as indicated.

*Data Collection and Case Definitions*

Sociodemographic (age, sex, self-reported race, and insurance status), comorbidities, and acute COVID-19 data (disease onset, severity, and treatments <4 weeks of disease onset) were collected by study physicians during the in-person visit and confirmed by electronic health record review. COVID-19 vaccination data were not collected systematically, so this data point was excluded from our analysis. As outpatient oxygen saturation stratification was not consistently available for mild acute COVID-19 cases, we defined mild cases as acute COVID-19 not requiring in-patient hospitalization within 14 days of symptom onset and severe cases as requiring in-patient hospitalization or intensive care unit (ICU) admission for COVID-19 complications within 14 days of symptom onset. Standard of care treatment for severe acute COVID-19 with SpO2 ≤ 94% during the course of this study included Remdesivir 200 mg IV loading dose on day 1 followed by 100 mg IV maintenance dose daily for 5 days and Dexamethasone 6 mg IV daily for 10 days or until hospital discharge. Remdesivir and corticosteroid regimens prescribed to mild acute COVID-19 participants did not follow a standardized protocol and occurred at the discretion of the clinical provider. Mild acute COVID-19 patients with ≥ 1 risk factors were eligible for monoclonal antibody treatment beginning August 2021. PASC duration was quantified as months from incident COVID-19 onset and analyzed in 3-month intervals (0-3, 3-6, 6-9, 9-12, ≥12 months).

New symptoms or worsening symptoms since COVID-19 onset persisting ≥3 weeks were collected using a standardized review of systems with dichotomous scoring (presence/absence) and were confirmed by clinician interview (Appendix I). To estimate severity of the most prevalent PASC symptoms in our clinic, participants completed validated patient-reported outcome (PRO) tools including the PROMIS Cognitive Function 8A (16), PROMIS Dyspnea 10A (17), PROMIS Fatigue 7A(18), Patient Health Questionnaire (PHQ-9) (19), and Generalized Anxiety Disorder (GAD-7) scales (20). Study data were collected and managed using REDCap electronic data capture tools hosted at Emory University (21).

*Statistical Analysis*

Sociodemographic and clinical characteristics are presented as absolute values and percentages for categorical values or medians and interquartile ranges (IQR) for continuous variables. Chi-square or Fisher’s exact tests were calculated to assess differences in categorical variables between groups where appropriate. PROMIS Patient-reported outcome measures were analyzed by comparing the proportion of respondents scoring ≥1.5 standard deviations (SD) from the mean. PHQ-9 and GAD-7 were analyzed comparing the proportion of respondents scoring ≥10, indicating moderate-severe disease on population-based norms. Univariable logistic regression was conducted to estimate odds ratios (OR) and 95% confidence intervals (CI) for associations between PASC symptoms or PROs and PASC duration intervals. Multivariable logistic regression analysis was used to estimate OR and 95% CI for association between clinical variables and PASC symptoms or PROs. Two-tailed p-values ≤0.05 were considered significant. We included participants for whom all variables of interest were available. No imputation was employed for missing data. Analysis was performed using SAS 9.4.

**Results**

*Study population*

During the study period, 332 participants enrolled (Table 1). The median age was 52 years (IQR 42-62), 233 (70%) were female, and more than half were self-reported African American/Black (172,52%), followed by Caucasian/White (103, 31%), Hispanic (15, 5%), Asian (13,4%), American Indian/Alaskan (2, <1%), and (2, <1%) Pacific Islander/Native Hawaiian. Median PASC duration was 107 days (IQR 60-191), and 145 (44%) participants presented between 3 weeks-3 months from incident infection, 101 (30%) between 3-6 months, 42 (13%) between 6-9 months, 26 (8%) between 9-12 months, and 18 (5%) greater than 12 months. The median Charleston comorbidity index (CCI) was 2 (IQR 1-4). As a proxy for socioeconomic status, 110 (33%) were uninsured or Medicaid recipients. Antecedent COVID-19 infections were mild in 171 (52%) and severe in 161 (48%). Of the severe infections, median length of hospital stay was 8 days (IQR: 5-14), and 40 (25%) were admitted to the intensive care unit (ICU). Mechanical ventilation was required in 18/40 (45%) of those admitted to the ICU.

**Table 1. Sociodemographic and Clinical Characteristics in Participants with Post-acute Sequelae of SARS-CoV-2 — August 2020–December 2021**

| **Characteristics** |  | **Acute COVID-19 Severity** | | **PASC Duration, months** | | | | |
| --- | --- | --- | --- | --- | --- | --- | --- | --- |
|  | **All participants** | **Mild** | **Severe** | **0-3** | **3-6** | **6-9** | **9-12** | **≥12** |
|  | **N= 332** | **N=171** | **N=161** | **N=145** | **N=101** | **N=42** | **N=26** | **N=18** |
|  | **(column %)** | **(col %)** | **(col %)** | **(col %)** | **(col %)** | **(col %)** | **(col %)** | **(col %)** |
| Age, median (IQR) | 52 (42-62) | 47 (39-56) | 59 (50-67) | 54 (41-64) | 53 (43-62) | 51 (47-59) | 51 (43-56) | 47 (39-64) |
| Female | 233 (70) | 132 (77) | 100 (63) | 100 (69) | 70 (69) | 33 (79) | 16 (62) | 14 (78) |
| Race |  |  |  |  |  |  |  |  |
| AI or AN | 2 (<1) | 1 (<1) | 1 (<1) | 2 (1) | 0 | 0 | 0 | 0 |
| Asian | 13 (4) | 7 (4) | 6 (4) | 5 (3) | 6 (6) | 2 (5) | 0 | 0 |
| Black or AA | 172 (52) | 68 (40) | 108 (65) | 86 (60) | 49 (49) | 21 (50) | 11 (42) | 5 (28) |
| Hispanic | 15 (5) | 6 (4) | 9 (6) | 5 (4) | 5 (5) | 1 (2) | 3 (12) | 1 (6) |
| NH or Other PI | 1 (<1) | 1 (<1) | 0 | 1 (<1) | 0 | 0 | 0 | 0 |
| White | 103 (31) | 66 (39) | 37 (23) | 38 (26) | 37 (36) | 13 (31) | 7 (27) | 8 (44) |
| Other/Unknown | 25 (8) | 22 (13) | 3 (2) | 7 (5) | 4 (4) | 5 (12) | 6 (19) | 4 (22) |
| Insurance |  |  |  |  |  |  |  |  |
| Uninsured | 74 (22) | 39 (23) | 35 (22) | 33 (45) | 20 (20) | 10 (24) | 8 (31) | 3 (17) |
| Medicaid | 38 (12) | 20 (12) | 18 (11) | 15 (10) | 11 (11) | 6 (15) | 3 (12) | 3 (17) |
| Medicare | 41 (12) | 8 (5) | 32 (20) | 17 (12) | 14 (14) | 5 (12) | 3 (12) | 2 (11) |
| Private | 172 (52) | 98 (58) | 74 (46) | 78 (54) | 52 (52) | 20 (49) | 12 (46) | 10 (56) |
| CCI, median (IQR) | 2 (1-4) | 2 (1-3) | 3 (2-5) | 3 (1-4) | 2 (1-4) | 3 (1-4) | 2 (1-4) | 2 (1-4) |
| Acute COVID-19 Severity |  |  |  |  |  |  |  |  |
| Asymptomatic | 1 (<1) | 1 (<1) | 0 | 0 | 1 (1) | 0 | 0 | 0 |
| Not hospitalized | 170 (51) | 170 (99) | 0 | 63 (44) | 55 (54) | 25 (60) | 17 (65) | 10 (56) |
| Hospitalized | 120 (36) | 0 | 120 (75) | 61 (42) | 33 (33) | 16 (38) | 5 (19) | 5 (28) |
| ICU | 40 (12) | 0 | 40 (25) | 20 (14) | 12 (12) | 1 (2) | 4 (15) | 3 (17) |
| Acute COVID-19 Treatment |  |  |  |  |  |  |  |  |
| Remdesivir | 114 (34) | 4 (2) | 110 (69) | 62 (43) | 28 (28) | 12 (29) | 8 (31) | 4 (22) |
| Corticosteroids | 184 (55) | 53 (31) | 131 (82) | 96 (66) | 51 (51) | 17 (41) | 11 (42) | 9 (50) |
| Anticoagulation | 128 (39) | 11 (6) | 117 (73) | 69 (48) | 34 (34) | 10 (24) | 9 (35) | 6 (33) |
| Monoclonal Antibodies | 18 (5) | 6 (4) | 12 (8) | 13 (9) | 3 (3) | 2 (5) | 0 | 0 |
| Plasma exchange | 7 (2) | 0 | 7 (4) | 6 (4) | 0 | 1(2) | 0 | 0 |
| PASC Duration, median days (IQR) | 107 (60-191) | 134 (65-209) | 90 (59-146) | 56 (45-71) | 121 (107-153) | 210 (193-230) | 310 (250-337) | 416 (385-463) |

*PASC Risk Factors*

The most common symptoms were dyspnea (n=236, 71%), fatigue (n=199, 60%), subjective cognitive impairment (n=156, 47%), cough (n=98, 30%), dizziness (n=73, 22%), and headache (n=69, 21%) (Table 2). Severe fatigue (n=76/263, 23%) and severe subjective cognitive impairment (n=60/259, 18%) defined as scoring >1.5 SD on PROMIS scales were common, whereas severe dyspnea (n=9/253, 3%) was infrequently reported. New or worsening moderate-severe depression and anxiety were seen in 26% and 16%, respectively. In adjusted models, mild acute COVID-19 severity was associated with higher rates of fatigue (OR: 1.83, 95% CI: 1.01-3.31), subjective cognitive impairment (OR: 2.76, 95% CI: 1.53-5.00), headaches (OR: 2.15, 95% CI: 1.05-4.44), and dizziness (OR: 2.41, 95% CI: 1.18-4.92) in our population (Figure 1). Additionally, they were less likely to demonstrate severe dyspnea, defined as scoring >1.5 SD on the PROMIS Dyspnea Scale (OR: 0.03, 95% CI: 0.002-0.47). No association was seen between COVID-19 severity and other reported symptoms. Similarly, longer PASC duration was associated with fatigue (OR: 1.54, 95% CI: 1.22-1.94), subjective cognitive impairment (OR: 1.47, 95% CI: 1.19-1.81), headaches (OR: 1.50, 95% CI: 1.18-1.90), severe fatigue (OR: 1.73, 95% CI: 1.35-2.22), and severe subjective cognitive impairment (OR: 1.32, 95% CI: 1.01-1.72).

**Table 2. Multivariable Logistic Regression Analysis of PASC Symptoms by Acute COVID-19 Severity**

|  |  | **Acute COVID-19 Severity** | |  |  |
| --- | --- | --- | --- | --- | --- |
| **Symptoms and Outcomes** | **All participants** | **Mild** | **Severe** | **OR (95% CI)** | **p-value** |
|  | **N= 332** | **N=171** | **N=161** |  |  |
| Dyspnea | 236 (71) | 125 (73) | 110 (69) | 0.98 (0.53-1.80) | 0.32 |
| Fatigue | 199 (60) | 117 (68) | 81 (51) | 1.83 (1.01-3.31)* | 0.047* |
| Cognitive Impairment | 156 (47) | 101 (59) | 55 (34) | 2.76 (1.53-5.00)* | 0.001* |
| Dizziness | 73 (22) | 49 (29) | 24 (15) | 2.41 (1.18-4.92)* | 0.02* |
| Headache | 69 (21) | 48 (28) | 21 (13) | 2.15 (1.05-4.44)* | 0.04* |
| Cough | 98 (30) | 49 (29) | 48 (30) | 1.34 (0.71-2.54) | 0.37 |
| Muscle pain | 61 (18) | 36 (21) | 25 (16) | 0.85 (0.38-1.89) | 0.69 |
| Anxiety | 60 (18) | 40 (23) | 19 (12) | 1.55 (0.73-3.31) | 0.25 |
| Depression | 52 (16) | 24 (14) | 28 (18) | 0.49 (0.22-1.10) | 0.09 |
| Joint pain | 53 (16) | 34 (20) | 19 (12) | 1.12 (0.48-2.59) | 0.78 |
| Palpitations | 53 (16) | 36 (21) | 17 (11) | 1.47 (0.60-3.61) | 0.40 |
| Weakness | 43 (13) | 16 (9) | 27 (17) | 0.66 (0.29-1.51) | 0.32 |
| Sleep disturbances | 40 (12) | 21 (12) | 19 (12) | 0.99 (0.44-2.22) | 0.97 |
| Anosmia | 38 (11) | 21 (17) | 17 (11) | 0.61 (0.23-1.66) | 0.34 |
| Dysgeusia | 50 (15) | 27 (16) | 23 (14) | 0.76 (0.32-1.878) | 0.53 |
| PROMIS Dyspnea >1.5 SD | 9/253 (3) | 1 (<1) | 8 (5) | 0.03 (0.002-0.47)* | 0.01* |
| PROMIS Fatigue >1.5 SD | 76/263 (23) | 47 (27) | 28 (17) | 1.14 (0.55-2.33) | 0.73 |
| PROMIS Cognitive >1.5 SD | 60/259 (18) | 41 (24) | 18 (11) | 2.13 (0.97-4.69) | 0.60 |
| PHQ-9 ≥10 | 85/263 (26) | 45 (26) | 39 (24) | 0.75 (0.44-1.28) | 0.29 |
| GAD-7 ≥10 | 5/127 (16) | 29 (17) | 24 (15) | 1.51 (0.62-2.89) | 0.46 |

*P<0.05 statistically significant

**Figure 1. Factors Associated with Prevalent PASC Symptoms**

Multivariable analysis of factors associated with the most prevalent PASC symptoms (A) fatigue, (B) subjective cognitive impairment, (C) headache, (D) dizziness, (E) dyspnea, and (F) cough, including Charleston Comorbidity Index (CCI), race, age, sex, PASC duration, acute COVID-19 severity, and acute COVID-19 treatments such as corticosteroids, monoclonal antibodies (MAB), anticoagulation, remdesivir. Sex reference value is female and acute COVID-19 severity reference is mild, as previously defined.

Women reported more fatigue (OR: 1.83, 95% CI: 1.01-3.31), headaches (OR: 2.68, 95% CI: 1.26-5.71), and cognitive impairment (OR: 1.77, 95% CI: 1.04-3.00) compared with men, and the perceived cognitive impairment was five times more severe in women (OR: 5.65, 95% CI: 2.00-16.0). Advanced age was modestly associated with fatigue (OR: 1.03, 95% CI: 1.00-1.06). Private insurance coverage (OR: 2.41, 95% CI: 1.94-3.00) and white race (OR: 1.61, 95% CI: 1.33-1.95) were associated with severe anxiety but had no effect on other symptoms. Those reporting cough were more likely to have received corticosteroids (OR: 2.15, 95% CI: 1.20-3.87) during acute infection. All other factors not stated were non-contributory.

*Impact of acute COVID-19 treatment*

Acute COVID-19 treatment with remdesivir was associated with less fatigue (OR: 0.47, 95% CI: 0.26-0.86) and fewer reporting severe symptoms (>1.5 SD) on the PROMIS Fatigue (OR: 0.47, 95% CI: 0.23-0.96) and PROMIS Cognitive Function scales (OR: 0.43, 95% CI: 0.20-0.92). This effect was not seen in other symptoms and severity scales.

*Temporal Profiles*

Fatigue prevalence was highest in those evaluated at 3-6 months from acute COVID-19 onset and persisted over time (Figure 2). These rates were highest beyond 3 months (OR: 3.29, 95% CI: 2.08-5.20) (Table 3). Similarly, cognitive impairment rates were significantly higher after 3 months (OR: 2.62, 95% CI: 1.67-4.11), but rates continued to increase over time. Despite this inflection point of 3-6 months for symptom report, PROMIS scales demonstrated that severe fatigue rates were highest after 9 months (OR: 3.85, 95% CI: 1.92-7.70) and continued to increase over time, and severe cognitive impairment rates increased significantly at 6-9 months (OR: 2.61, 95% CI: 1.29-5.27) and remained elevated with highest rates occurring after 6 months (OR: 2.90, 95% CI: 1.58-5.33). Headaches spiked in prevalence at 9-12 months (OR: 5.80, 95% CI: 1.94-17.3) followed by a decline towards previous rates. There were no significant changes in prevalence of other symptoms across PASC durations studied, though dizziness and cough had a trend towards improvement with time.

**Figure 2. Frequency of (A) Prevalent PASC Symptoms and (B) Symptom-specific Severity Outcomes across PASC Duration.** (A) Fatigue, subjective cognitive impairment, headache, dizziness, dyspnea, and cough and (B) Frequency of scores ≥1.5 standard deviations from the mean on PROMIS Fatigue, PROMIS Cognitive, and PROMIS Dyspnea were analyzed across 3-month intervals from incident infection (T_0_): 0-3, 3-6, 6-9, 9-12, and ≥12 months.

**Table 3. PASC Duration Intervals Associated with Prevalent Symptoms and Symptom-specific Severity Outcomes**

| **Symptoms and Outcomes** | **PASC Duration Intervals, months** | | | | | |
| --- | --- | --- | --- | --- | --- | --- |
|  | **3-6 vs 0-3** | **6-9 vs 3-6** | **9-12 vs 6-9** | **3-12+ vs 0-3** | **6-12+ vs 0-6** | **9-12+ vs 0-9** |
|  | **OR (95% CI)** | **OR (95% CI)** | **OR (95% CI)** | **OR (95% CI)** | **OR (95% CI)** | **OR (95% CI)** |
| Symptoms |  |  |  |  |  |  |
| Fatigue | 3.30 (1.91-5.69)* | 0.86 (0.39-1.88) | 1.49 (0.49-4.59) | 3.29 (2.08-5.20)* | 2.06 (1.20-3.51)* | 2.21 (1.07-4.54)* |
| Cognitive Impairment | 2.16 (1.29-3.64) | 1.33 (0.64-2.76) | 1.28 (0.47-3.55) | 2.62 (1.67-4.11)* | 2.38 (1.44-3.95)* | 2.45 (1.26-4.77)* |
| Dizziness | 2.14 (1.15-3.97) | 0.99 (0.45-2.20) | 0.60 (0.18-1.94) | 1.94 (1.11-3.36)* | 1.21 (0.68-2.15) | 0.90 (0.41-1.97) |
| Headache | 1.85 (0.94-3.63) | 0.34 (0.34-2.09) | 5.80 (1.94-17.3)* | 2.42 (1.35-4.33)* | 2.41 (1.38-4.23)* | 4.07 (2.08-7.93)* |
| Dyspnea | 0.84 (0.49-1.46) | 1.55 (0.68-3.54) | 1.04 (0.33-3.31) | 1.00 (0.62-1.62) | 1.36 (0.77-2.38) | 1.26 (0.61-2.60) |
| Cough | 1.07 (0.62-1.84) | 1.08 (0.50-2.32) | 0.60 (0.20-1.83) | 0.93 (0.58-1.50) | 0.77 (0.44-1.34) | 0.49 (0.22-1.09) |
| Symptom Severity Outcomes |  |  |  |  |  |  |
| PROMIS Dyspnea >1.5 SD | 0.70 (0.39-1.24) | 1.64 (0.75-3.57) | 0.38 (0.11-1.30) | 5.71 (0.70-46.3) | 1.36 (0.33-5.58) | 0.68 (0.08-5.60) |
| PROMIS Fatigue >1.5 SD | 0.77 (0.46-1.29) | 1.82 (0.91-3.62) | 0.92 (0.37-2.31) | 2.58 (1.43-4.68)* | 3.20 (1.80-5.68)* | 3.85 (1.92-7.70)* |
| PROMIS Cognitive >1.5 SD | 0.60 (0.35-1.02) | 2.61 (1.29-5.27)* | 0.50 (0.19-1.29) | 1.76 (0.95-3.27) | 2.90 (1.58-5.33)* | 2.04 (0.99-4.21) |
| PHQ-9 ≥10 | 0.68 (0.42-1.12) | 1.68 (0.83-3.27) | 0.75 (0.30-1.90) | 0.77 (0.51-1.17) | 1.06 (0.66-1.69) | 0.86 (0.46-1.59) |
| GAD-7 ≥10 | 1.14 (0.69-1.89) | 1.03 (0.50-2.11) | 0.78 (0.30-2.02) | 1.18 (0.77-1.82) | 1.17 (0.71-1.92) | 1.21 (0.63-2.30) |

*P<0.05 statistically significant

**Discussion**

In this study, participants with mild acute COVID-19 and longer PASC durations had increased prevalence of common PASC symptoms. This is a shift in the current theoretical framework that assumes a direct relationship between acute phase severity and PASC development. Although early studies have described higher prevalence of PASC in those with severe acute COVID-19 (11), when controlling for confounders, this association remains equivocal. In previous studies, no association was found in the first 3 months following infection (12, 13), while others found higher prevalence of fatigue, weakness, depression, and anxiety at 6 months following severe acute COVID-19, with no differences in rates by 1 year (3, 7). In contrast, here we describe higher prevalence of fatigue, cognitive impairment, headaches, and dizziness in those with mild antecedent COVID-19. With mild disease comprising 94% of COVID-19 cases and PASC occurring in a substantial proportion of survivors, our findings have important policy implications (9, 22, 23). Vaccination campaigns and masking strategies primarily focus on mitigating risk of acute COVID-19 morbidity and mortality, but our findings underscore the importance of preventing mild disease given these known, debilitating sequelae. This effect may have gone undetected previously as few studies have analyzed risk factors associated with independent PASC symptoms (3, 10, 12, 13). Interestingly, dyspnea severity had an opposite association wherein it was directly correlated with severe acute COVID-19. Further investigation is needed to determine if distinct phenotypes exist within PASC that prevent analysis of symptoms in aggregate.

Our study design has the advantage of characterizing symptom profiles at multiple PASC intervals, including longer intervals which have not been well characterized. Complaints of fatigue, cognitive impairment, and headache occurred at higher prevalence in those with longer PASC duration. In fact, a PASC duration of 3-6 months appears to be a breakpoint at which fatigue becomes more prevalent and persists in our population. Similarly, prevalence of subjective cognitive impairment rapidly increase at 3-6 months followed by a steady increase over time. Although there is still much to learn about PASC symptom clusters, this supports demarcation of a distinct, delayed phenotype that, for some, may not improve with time. Interestingly, our study showed an association with remdesivir, an anti-viral which may provide some protection against these symptoms. This delayed phenotype overlaps with a symptom cluster and longitudinal course described previously (6), but differs from others who describe higher fatigue beyond one year in PASC with no change in cognitive symptoms over time (10). Additionally, we found that fatigue and cognitive impairment severity continues to increase over time with severity escalating after 9 months for fatigue and after 6 months for cognitive impairment (10). Characterization of cognitive impairment and severity is key to this phenotype and is an important element of this study as neurocognitive effects are among the most prevalent PASC symptoms but are-understudied in the current literature (3, 4, 6, 7, 13, 24). The temporal pattern for headaches appears unique from fatigue and cognitive impairment with headache prevalence peaking at 9-12 months, followed by quick decline. Dizziness prevalence increased and remained elevated in those 3-9 months post incident SARS-CoV-2 infection followed by improvement later in PASC course, consistent with previous reports (6). Similarly, prevalence of cough declined over time. Although prevalence of dyspnea remains static, severity of symptoms may continue to increase over time, consistent with previous findings showing higher prevalence at 12 months (10).

In our study, receipt of remdesivir during the acute phase was associated with less fatigue and decreased severity of fatigue and subjective cognitive impairment. Antivirals have not shown benefit previously; however, prior analyses investigated composite antivirals, including drugs with diverse mechanisms such as hydroxychloroquine, which has shown no significant effect in the acute COVID-19 outcomes (3, 7, 25). The mechanism by which remdesivir may impact fatigue and cognitive impairment is unclear. Though studies have failed to show significant nasopharyngeal viral load reduction in SARS-CoV-2 infected individuals following remdesivir treatment (26), the potential protective effects against this symptom profile may support the theory of a persistent viral reservoir that triggers ongoing immune responses, contributing to the underlying pathology of PASC (27, 28). Further studies are needed to determine if remdesivir interrupts this cascade through viral clearance in other tissues that are not accurately represented by nasopharyngeal sampling. Perhaps the use of anti-viral treatments in COVID-19 is reminiscent of antimicrobial treatment in group A streptococcus infections, wherein early treatment provides protection against post-acute sequelae affecting multiple organ systems(29). Remdesivir use showed no association with development or persistence of dyspnea or cough, and there was no significant temporal pattern for these symptoms, which may indicate a heterogenous underlying mechanism in those experiencing these symptoms. This is consistent with the previously described cluster phenotype, which excluded pulmonary symptoms (6).

Female PASC patients were more prone to fatigue, cognitive impairment, and headaches than male counterparts. Women have been reported to have higher rates of composite PASC symptoms (24), though, adjusted models have found either no association (13) or symptom-specific associations with fatigue, headache, anxiety and depression (3, 22). Though advanced age has been identified as a risk factor for PASC development (11, 24), age was not a significant factor for individual symptoms in previous adjusted models(3, 13, 14), with the exception of a modest effect of higher fatigue in older patients in a previous cohort (3) as well as our own. Similarly, minority race has been shown to contribute higher morbidity and mortality from acute COVID-19 (30); however, race has not remained significant in adjusted PASC models (14). Our study included a diverse population with broad socioeconomic representation, and we report similar findings, only observing a sociodemographic effect for white, privately insured participants who experienced more severe anxiety.

There are several limitations of this study. As this study exclusively enrolled PASC patients presenting to care, symptom prevalence is overestimated compared with the general population. Additionally, this design is subject to referral bias and may skew towards a PASC population with higher symptom burden and severity. Reported symptom percentages represent symptom burden in a population of PASC patients referred for specialty care and are presented to demonstrate relative proportions between groups to study contributory and mitigating factors associated with symptom profiles. Limiting enrollment to PASC clinic patients risks exclusion of historically marginalized groups including those with financial limitations and poor access to care; however, 34% of enrollees were uninsured or Medicaid recipients. During the study period, our PASC clinic referral queue exceeded our ability to schedule standardized follow-up visits and many patients were lost to follow-up. As such our study does not provide longitudinal assessments with multiple PASC duration time points per patient. Rather, our PASC duration groups represent distinct patient populations. Participant characteristics did not differ significantly between PASC duration groups, with the exception of higher proportion of black race in those with shorter PASC duration; however, race did not contribute significantly in adjusted models. Given the observational nature of this study, the associations described between remdesivir use and decreased fatigue and cognitive impairment should be interpreted with caution as there may be additional confounding factors not accounted for in the study design. To better inform this relationship, remdesivir could be considered for future randomized clinical trials.

In this study, we demonstrate that mild acute COVID-19 and PASC duration are associated with fatigue, subjective cognitive impairment, headaches, and dizziness and that those treated with remdesivir in the acute phase experienced lower rates and severity of fatigue as well as less severity of cognitive impairment. Though the pathobiology of PASC remains unknown, these findings provide some insight into potential physiologic mechanisms and targets for prevention of particular PASC phenotypes. Furthermore, we show that these symptoms may have a delayed onset ranging 3-12 months from incident infection, and a subset of these patients will continue to have persistent symptoms that do not improve significantly over time, underscoring the importance for targeted COVID-19 preventative measures.

*Potential Conflicts of Interests*. Dr. F. Eun-Hyung Lee reports grants from Genetech and the Gates Foundation. She has received royalties for BLI, Inc and consulting fees from Be Bio Pharma. She received honoraria from Gerontological Advanced Practice Nurses Association and has patents for plasma cell survival media and MENSA. She is the founder of the MicroB-plex, Inc. Dr. Ignacio Sanz reports grants from GlaxoSmithKline, Bristol Myers Squibb, Exagen, and consulting fees from Pfizer, Octagon, and Bristol Myers Squibb. He serves on the DSMB for and has stock options in Kyverna.

*Funding*. This work was supported by the Woodruff Health Science Center COVID-19 CURE Award through philanthropic support from the O. Wayne Rollins Foundation and the William Randolph Hearst Foundation and through in-kind support from Grady Healthcare. The funding source has no role in study design, collection, analysis or interpretation of data, writing of reports, nor decision to submit papers for publication. The findings and conclusions in this report are those of the authors and do not necessarily represent the official position of the Woodruff Health Science Center, Emory University, Morehouse School of Medicine, Grady Memorial Hospital, or affiliated partners.

*Data sharing*

All the relevant data are in the within the manuscript and its supporting information files.

References

1.	COVID-19 Dashboard [Available from: <https://coronavirus.jhu.edu/map.html>.

2.	Gupta A, Madhavan MV, Sehgal K, Nair N, Mahajan S, Sehrawat TS, et al. Extrapulmonary manifestations of COVID-19. Nat Med. 2020;26(7):1017-32.

3.	Huang C, Huang L, Wang Y, Li X, Ren L, Gu X, et al. 6-month consequences of COVID-19 in patients discharged from hospital: a cohort study. Lancet. 2021;397(10270):220-32.

4.	Garrigues E, Janvier P, Kherabi Y, Le Bot A, Hamon A, Gouze H, et al. Post-discharge persistent symptoms and health-related quality of life after hospitalization for COVID-19. J Infect. 2020;81(6):e4-e6.

5.	Xiong Q, Xu M, Li J, Liu Y, Zhang J, Xu Y, et al. Clinical sequelae of COVID-19 survivors in Wuhan, China: a single-centre longitudinal study. Clin Microbiol Infect. 2021;27(1):89-95.

6.	Davis HE, Assaf GS, McCorkell L, Wei H, Low RJ, Re'em Y, et al. Characterizing long COVID in an international cohort: 7 months of symptoms and their impact. EClinicalMedicine. 2021;38:101019.

7.	Huang L, Yao Q, Gu X, Wang Q, Ren L, Wang Y, et al. 1-year outcomes in hospital survivors with COVID-19: a longitudinal cohort study. Lancet. 2021;398(10302):747-58.

8.	Petersen MS, Kristiansen MF, Hanusson KD, Danielsen ME, B ÁS, Gaini S, et al. Long COVID in the Faroe Islands - a longitudinal study among non-hospitalized patients. Clin Infect Dis. 2020.

9.	Logue JK, Franko NM, McCulloch DJ, McDonald D, Magedson A, Wolf CR, et al. Sequelae in Adults at 6 Months After COVID-19 Infection. JAMA Netw Open. 2021;4(2):e210830.

10.	Seeßle J, Waterboer T, Hippchen T, Simon J, Kirchner M, Lim A, et al. Persistent Symptoms in Adult Patients 1 Year After Coronavirus Disease 2019 (COVID-19): A Prospective Cohort Study. Clin Infect Dis. 2022;74(7):1191-8.

11.	Carvalho-Schneider C, Laurent E, Lemaignen A, Beaufils E, Bourbao-Tournois C, Laribi S, et al. Follow-up of adults with noncritical COVID-19 two months after symptom onset. Clin Microbiol Infect. 2021;27(2):258-63.

12.	Townsend L, Dyer AH, Jones K, Dunne J, Mooney A, Gaffney F, et al. Persistent fatigue following SARS-CoV-2 infection is common and independent of severity of initial infection. PLoS One. 2020;15(11):e0240784.

13.	Moreno-Pérez O, Merino E, Leon-Ramirez JM, Andres M, Ramos JM, Arenas-Jiménez J, et al. Post-acute COVID-19 syndrome. Incidence and risk factors: A Mediterranean cohort study. J Infect. 2021;82(3):378-83.

14.	Jacobson KB, Rao M, Bonilla H, Subramanian A, Hack I, Madrigal M, et al. Patients With Uncomplicated Coronavirus Disease 2019 (COVID-19) Have Long-Term Persistent Symptoms and Functional Impairment Similar to Patients with Severe COVID-19: A Cautionary Tale During a Global Pandemic. Clin Infect Dis. 2021;73(3):e826-e9.

15.	A clinical case definition of post COVID-19 condition by a Delphi consensus 6 October 2021 [Available from: <https://www.who.int/publications/i/item/WHO-2019-nCoV-Post_COVID-19_condition-Clinical_case_definition-2021.1>.

16.	PROMIS Short Form v2.0 - Cognitive Function Abilities Subset 8a 02Jan2020 [Available from: <https://www.healthmeasures.net/index.php?option=com_instruments&view=measure&id=769&Itemid=992>.

17.	PROMIS Short Form v1.0 - Dyspnea Severity 10A 02Sept2016 [Available from: <https://www.healthmeasures.net/index.php?option=com_instruments&view=measure&id=770&Itemid=992>.

18.	PROMIS Short Form v1.0 - Fatigue - 7a 31July2018 [Available from: <https://www.healthmeasures.net/index.php?option=com_instruments&view=measure&id=160&Itemid=992>.

19.	Kroenke K, Spitzer RL, Williams JB. The PHQ-9: validity of a brief depression severity measure. J Gen Intern Med. 2001;16(9):606-13.

20.	Löwe B, Decker O, Müller S, Brähler E, Schellberg D, Herzog W, et al. Validation and standardization of the Generalized Anxiety Disorder Screener (GAD-7) in the general population. Med Care. 2008;46(3):266-74.

21.	Harris PA, Taylor R, Thielke R, Payne J, Gonzalez N, Conde JG. Research electronic data capture (REDCap)--a metadata-driven methodology and workflow process for providing translational research informatics support. J Biomed Inform. 2009;42(2):377-81.

22.	Taquet M, Dercon Q, Luciano S, Geddes JR, Husain M, Harrison PJ. Incidence, co-occurrence, and evolution of long-COVID features: A 6-month retrospective cohort study of 273,618 survivors of COVID-19. PLoS Med. 2021;18(9):e1003773.

23.	CDC COVID-19 Data Tracker [Available from: <https://covid.cdc.gov/covid-data-tracker/#new-hospital-admissions>.

24.	Jacobs JJL. Persistent SARS-2 infections contribute to long COVID-19. Med Hypotheses. 2021;149:110538.

25.	Boulware DR, Pullen MF, Bangdiwala AS, Pastick KA, Lofgren SM, Okafor EC, et al. A Randomized Trial of Hydroxychloroquine as Postexposure Prophylaxis for Covid-19. N Engl J Med. 2020;383(6):517-25.

26.	Barratt-Due A, Olsen IC, Nezvalova-Henriksen K, Kåsine T, Lund-Johansen F, Hoel H, et al. Evaluation of the Effects of Remdesivir and Hydroxychloroquine on Viral Clearance in COVID-19 : A Randomized Trial. Ann Intern Med. 2021;174(9):1261-9.

27.	Sun J, Xiao J, Sun R, Tang X, Liang C, Lin H, et al. Prolonged Persistence of SARS-CoV-2 RNA in Body Fluids. Emerg Infect Dis. 2020;26(8):1834-8.

28.	Gaebler C, Wang Z, Lorenzi JCC, Muecksch F, Finkin S, Tokuyama M, et al. Evolution of antibody immunity to SARS-CoV-2. Nature. 2021;591(7851):639-44.

29.	Cunningham MW. Pathogenesis of group A streptococcal infections. Clin Microbiol Rev. 2000;13(3):470-511.

30.	Mackey K, Ayers CK, Kondo KK, Saha S, Advani SM, Young S, et al. Racial and Ethnic Disparities in COVID-19-Related Infections, Hospitalizations, and Deaths : A Systematic Review. Ann Intern Med. 2021;174(3):362-73.
