# Supplementary material for: Mild antecedent COVID-19 associated with symptom-specific post-acute sequelae
Source: PLoS One. 2023 Jul 10;18(7):e0288391. doi: 10.1371/journal.pone.0288391 (PMC10332615; doi:10.1371/journal.pone.0288391)
Supplement: Attachment — Submitted filename: Response to Reviewers.docx [file pone.0288391.s005.docx]

**Response to Reviewers**

**Manuscript Text Reviewer Comments:**

1. Depending on time of publishing may need to update these numbers
a) Author response: Updated with current case and death counts in Line 51
2. Many of the studies you cite are among hospitalized patients – the prevalence of PASC among all individuals infected with SARS-CoV-2 is much lower so I would update this value

<https://pubmed.ncbi.nlm.nih.gov/34007978/>

<https://academic.oup.com/cid/article/73/11/2055/6276644>

<https://academic.oup.com/jid/article/226/9/1593/6569364?login=false>

a. Author response: I agree with this point. Included these citations as well as the Bull-Otterson MMWR: <https://www.cdc.gov/mmwr/volumes/71/wr/mm7121e1.htm> in Line 56

3. Are you distinguishing between symptoms that occur during acute infection that last for 3+ months and new symptoms that occur after initial infection? I see you mention persisting symptoms later in methods – feel free to disregard
   a. Author response: no edits suggested
4. Public health education or clinical education? It seems that the target audience of this paper is more clinical rather than geared towards public health practitioners
   a. Author response: Good point. I have made this suggested edit in Line 59
5. Concurrently?
   a. Author response: I chose consecutively to demonstrate that all patients presenting to our PASC clinics had an opportunity to enroll. We did not skip patients or select certain patients. I'm concerned that "concurrently" doesn't indicate this equity in sampling, but I am open to the change if the editor agrees with the suggested edit.
6. Should maybe mention how vaccination can potentially affect risk of PASC in discussion. Would potentially be a confounder for symptom severity and PASC outcome
   a. Author response: Good point. I have added a comment on vaccination impact on acute disease severity and PASC development to the discussion, Line 210 and limitations, Line 286.
7. I would make this consistent with your methods above, it was a little unclear to me in the earlier section
   a. Author response: Thank you. Edit addressed in Line 85.
8. For multivariable models how were parameters selected for inclusion? What metrics (ie AIC) were used to determine final model structure?
   a. Author response: Variables that were significant on univariate analysis and those with clinical relevance were incorporated into the multivariable model. AIC was used to determine goodness-of-fit, and variables optimizing AIC were retained. Edit addressed in the text in Line 121.
9. Acute symptom clinical variables? Clinical variables from enrollment at the PASC center?
   a. Author response: Clinical variables include: sociodemographic, comorbidity, acute COVID-19 severity and treatment variables. Edit is addressed in the text on Line 119.
10. Citation for software?
    a. Author response: Reference was added to the text, Line 125
11. Did you perform a sample size calculation?
    a. Author response: A sample size calculation was performed prior to study initiation, estimating 300 participants needed to achieve 90% power to detect changes in sequelae trajectories in longitudinal analysis. However, we were unable to attain repeated measures in a sufficient number of patients due to burden of new PASC patients filling appointments in our PASC clinics leaving limited appointments for follow-up patients, as described in the study limitations section. For this reason, we were unable to analyze longitudinal trends in symptomatology over time.
12. Did you combine race and ethnicity into one category? Please clarify in methods.
    a. Author response: Race and ethnicity were analyzed together as one variable in models. Edit is addressed in the text, Line 120
13. These sentences could potentially be combined, but not necessary if authors prefer to keep separate
    a. Author response: I agree. Edit is addressed in the text, Line 137
14. This should be months after incident infection – the current wording could indicate that participants only experience PASC for X duration. Changing wording to reflect time elapsed (months after incidence infection) can help make it clear that participants are experiencing PASC for at least X amount of time
    a. Author response: I have made this suggested edit in Table 1
15. Would be good to write out just so it’s easier for the reader to understand values at a glance
    a. Author response: Agree with edit made in the text in Table 1
16. What do you categorize as longer PASC?
    a. Author response: PASC duration was quantified as months from incident COVID-19 onset and analyzed in 3-month intervals (0-3, 3-6, 6-9, 9-12, ≥12 months). In models, PASC duration was positively correlated with fatigue, cognitive impairment, headaches, severe fatigue, and subjective cognitive impairment, indicating that those with longer PASC durations (those at the longer end of the spectrum) have higher odds of these symptoms. Clarification made in the text, Line 154
17. Reiterate that these are percentages
    a. Author response: I have made this suggested edit in Table 2.
18. I believe PLOS asks for 2 significant figures so double check all tables
    a. Author response: I have not seen guidance on significant digits, but I can revise if suggested by the editor. I have corrected the error noted in the text in Table 2.
19. What are you defining as advanced age?
    a. Author response: Updated the text to define advanced age as (≥65 years) in Line 170
20. Please clarify use of rate versus prevalence throughout. If you are only capturing new cases of PASC it is fine to use rate in reference to incidence but otherwise prevalence should be used. Since you used prevalence in L173 please use it consistently
    a. Author response: Updated the text to change “rate” to prevalence at all points.
21. Revise if this language is incorrect
    a. Author response: Text has been updated to use the language “prevalence”. No further edits needed.
22. Please use prevalence instead of rate throughout
    a. Author response: Edits made in the text as requested
23. Comment on how vaccination status can affect acute symptom severity and potentially PASC development
    a. Author response: I have added a comment on vaccination impact on acute disease severity and PASC development to the discussion, Line 210 and limitations, Line 286.
24. This sentences seems to imply tracking patients repeatedly but you indicated in methods that your study participants were only evaluated once. Instead of saying over time, switch focus to increase by time since incident infection
    a. Author response: Agree with this point as well. Please see amended text, Line 227
25. Same comment as before regarding time
    a. Author response: Agree with this point as well. Please see amended text, Line 234
26. I would double check the epidemiologic literature for this
    a. Author response: Please see suggested edits. I updated the text with current literature on age as a risk factor for individual PASC symptoms, Line 269
27. Please check some of these other epidemiological studies that do show an association between race/ethnicity and PASC.

<https://www.ncbi.nlm.nih.gov/pmc/articles/PMC8445372/>

<https://www.nature.com/articles/s41467-021-26513-3>

a. Author response: Thank you. When individual symptoms are analyzed, it appears that there may be symptoms that are more common in specific racial/ethnic groups. Many of the same symptoms we studied remained insignificant between racial groups in adjusted models in the Xie paper referenced (see Xie Supplementary Table 6), and some of these symptoms were found to be more common in white participants in the Khuller adjusted analysis. See suggested edits in the text, Line 274

**Reviewer 1 General comments:**

Reviewer #1: This manuscript presents novel evidence to our understanding of long COVID and development of post-acute sequelae among individuals with mild acute symptoms. I had a number of minor edits that I detail in the attached document, but I believe that after addressing my comments this paper should add key knowledge to the scientific literature on long COVID.

I would especially urge the authors to use prevalence instead of rate throughout the manuscript as to not confuse readers who may not understand the difference between prevalence and incidence.

a. Author response: Thank you for this suggestion. I have updated the text with this correction.

**Reviewer 2 General comments:**

Reviewer #2: This study analyzed the risk for each symptom of PASC, and also demonstrated the prevalence of symptom profiles over time to evaluate the disease course. Authors dealt with an interesting topic and drew valid conclusions through a thorough literature search and data analysis. However, I think some revisions are required.


1. Variant type of SARS-CoV-2 were changed during the study period. Didn't you check the effect of the variant? Even if the virus type cannot be determined through the PCR test, the analysis can be performed based on the time point when the omicron became the dominant variant.
a. Author response: The authors agree with this suggestion. Given the time span that the study was conducted, the majority (94%) of the infections were the original strain and the remaining 6% were presumed Delta (infections after 7/1/2021 and before 12/1/2021). There were no infections after 12/1/2021 in our cohort. We have provided a subgroup analysis as Appendix II demonstrating the effect of variant on symptom type. Most are not statistically significant, likely due to the small sample size for Delta; however, we did see less anxiety in alpha (more anxiety in Delta). The text has been updated: methods described definition (Line 88) of variants and subgroup analysis (Line 118), results reference Appendix II findings (136), discussion mentions limitation of not having more diversity of variants to evaluate effect of currently circulating strain (Line 297).

2. In this study, severe patients were not separated from critical patients. It would be desirable to demonstrate the PASC of critical patients by subgroup analysis.
a. Author response: Thank you for this suggestion. We conducted a subgroup analysis of critical acute COVID-19 patients and found that critical COVID-19 is associated with more dyspnea and less subjective cognitive impairment, joint pain, anosmia, and severity of fatigue and depression, consistent with general trends seen in the mild vs severe COVID-19 comparison. This population only comprises 12% of the study population and small sample size may affect ability to detect differences in other variables. The text has been updated in the results section, referencing the findings (Appendix III)- Line 175, and the discussion has been updated, Line 220. The methods section already defines critical COVID-19.

3. In Table 1, mild patients were persons who were not hospitalized, but how did they receive remdesivir. Did all these patients receive it outpatients?
a. Author response: Four patients received 3 days of outpatient infusions at an infusion center. This was off label and at the discretion of their provider. I have expanded the explanation in the methods section, Line 98

4. Since vaccination is an important factor that can affect the PASC, authors should describe the point that effect of vaccine was not analyzed as a limitation in the discussion section.

Author response: I have added a comment on vaccination impact on acute disease severity and PASC development to the discussion, Line 210 and limitations, Line 286.


1. Why do you think dexamethasone was a risk factor for persistent cough after COVID-19?

a. Author response: This is unclear. It could be due to the fact that those with chronic pulmonary diseases (COPD, asthma) were more likely to require dexamethasone treatment during a COVID-19 infection. The adjusted model analyzes comorbidities by the Charleston Comorbidity Index and not individual comorbidities. However, when I conduct a sensitivity analysis using COPD and asthma in place of the CCI, there remains a slight effect for dexamethasone and persistent cough. Potentially, there are participants with underlying pulmonary diseases that have gone undiagnosed, driving this effect. Explanation is added to the discussion, Line 263.
