# Supplementary material for: Mild antecedent COVID-19 associated with symptom-specific post-acute sequelae
Source: PLoS One. 2023 Jul 10;18(7):e0288391. doi: 10.1371/journal.pone.0288391 (PMC10332615; doi:10.1371/journal.pone.0288391)
Supplement: Attachment — Submitted filename: Response to Reviewers.docx [file pone.0288391.s006.docx]

**Response to Reviewers**

**Manuscript Text Reviewer Comments:**

1. The authors should include their description of the sample size calculation in the methods section. It was noted in response to reviewer’s comments

Author response: Edits made. Power calculation added to Statistical analysis Line 117*. Limitations in completing longitudinal analysis addressed previously in Line 309*

2. All abbreviation should initially spell out in main text and table/figure respectively. e.g. AIC in main text and AI, AA, CCI in table, and so on

Author response: Edits made. Abbreviations/acronyms have been spelled out in text, tables, and figures.

3. All tables and figures are showed in main text. However, I can’t find table 2 in main text.

Author response: Table 2 is present in Line 165* and referenced on Line 151-152*

4. The authors used 3 different supplementary materials. However, the authors used same number, supplementary appendix II, twice in table title. To avoid confusion, I suggest to use supplementary table 1, 2 and 3 in main text and title instead of appendix I and II

Author response: Edit made. All supplementary materials are now listed as Supplementary Table 1, 2, and 3 in the main text and supplement title.

5. In line 154-158, In which table or figure is this description presented?

Author response: All text in this paragraph is in reference to Figure 1, which is referenced in Line 158*. I have added an additional reference to Figure 1 in Line 160* for clarity.

6. Is it right for figure 2 title and legend? Please add a title of Y axis in table 2

Author response: Yes, the Figure 2 title and legend are correct. Title of Y axis has been added to table 2.

*Listed Line numbers refers to location in tracked-changes version
