# Supplementary material for: Mild antecedent COVID-19 associated with symptom-specific post-acute sequelae
Source: PLoS One. 2023 Jul 10;18(7):e0288391. doi: 10.1371/journal.pone.0288391 (PMC10332615; doi:10.1371/journal.pone.0288391)
Supplement: Attachment — Submitted filename: Response to Reviewers.docx [file pone.0288391.s007.docx]

**Response to Reviewers**

1. In line 187-190, the association between remdesivir and PASC symptoms is a key point in this paper. However, I cannot find in which table or figure this description was presented. Please check it again.
   a. Author response: These data are represented in Figure 1 and follow a paragraph that discusses data represented in Figure 1. I have not added additional reference to Figure 1 per your recommendation below, but would be happy to do so if needed for clarity.
2. It would be better to avoid repeating figure or table cited in main text. So, Figure 1 can be removed presenting in line 159.
   a. Author response: Duplicate reference to Figure 1 has been removed
